# The Relationship Between Long Working Hours and Stress Responses in Junior High School Teachers: A Nationwide Survey in Japan

**DOI:** 10.3389/fpsyg.2021.775522

**Published:** 2022-01-11

**Authors:** Masateru Matsushita, Schuhei Yamamura

**Affiliations:** ^1^Department of Psychology, Faculty of Human Sciences, Konan Women’s University, Kobe, Japan; ^2^Department of Psychiatry, Kinki Central Hospital of the Mutual Aid Association of Public School Teachers, Itami, Japan

**Keywords:** occupational health, mental health, stress response, overtime work, school teachers, job title

## Abstract

**Background:** Long working hours and mental health problems among teachers are a concern in Japan. More specifically, it has been reported that junior high school teachers tend to work overtime. In this study, examined the working hours of junior high school teachers in public schools and investigated the association between overtime work and stress responses across job titles.

**Methods:** From June to December 2018, 54,772 teachers in public junior high schools completed a web-based nationwide survey regarding occupational stress and submitted self-evaluated working hours per day of the previous month. Psychological and physical stress responses were assessed using the Brief Job Stress Questionnaire.

**Results:** Results showed that 59.6% of the participants worked 11 h or more per day. Additionally, the length of working hours significantly differed across job titles (χ^2^ (30) = 5295.8, *p* < 0.001, Cramér’s *V* = 0.14). With respect to tenured teachers, sex (female), age, taking charge of the class, number of working years in the same school, working hours of 10 to 11 h, 11 to 12 h, 12 to 13 h, and 13 h or more were significantly associated with high stress, compared to those who worked less than 9 h per day. Moreover, for fixed-term teachers, sex (female), age, working hours of 9 to 10 h, 10 to 11 h, 11 to 12 h, 12 to 13 h, and 13 h or more were related with more stress as compared to those who worked less than 9 h per day. On the other hand, there was no significant relationship between long working hours and stress response among vice-principals, even though they tended to work the longest hours.

**Conclusion:** We verified that Japanese junior high school teachers work long hours. Long working hours were associated with stress responses in both tenured and fixed-term teachers, but not in vice-principals. However, vice-principals work the longest hours among teachers, and we suggest that these long working hours may be a hidden problem that is often overlooked.

## Introduction

In Japan, the percentage of public school teachers on leave from work due to mental disorders reached 0.59% in 2019, according to the Ministry of Education, Culture, Sports, Science and Technology (MEXT) (Ministry of Education, Culture, Sports, Science and Technology [MEXT], 2020). This was the second-highest percentage ever recorded and the number of teachers on leave has remained high in recent years. The MEXT analyzed related data and reported that these leaves of absence due to mental illness were caused by increased and more complicated workloads and by problems in interpersonal relationships in the workplace. Other possible causes of absence from work may be long working hours and workload (Ministry of Education, Culture, Sports, Science and Technology [MEXT], 2020). It is well known that teachers in Japan, especially those in junior high schools, work long hours (Organization for Economic Cooperation and Development [OECD], 2018). In 2018, the Teaching and Learning International Survey (TALIS) conducted by the OECD surveyed the working environment of secondary school teachers in 48 member countries and regions. It was reported that teachers in Japan work 56 h per week, the longest work week among the countries surveyed (Organization for Economic Cooperation and Development [OECD], 2018). In Japan, Article 32 of the Labor Standards Law stipulates that working hours should be no more than 8 h per day and 40 h per week (Ministry of Justice, Japan, 2012). Work performed beyond these working hours is considered overtime. The upper limit for overtime work is 360 h per year, which cannot be exceeded unless there are special circumstances. In addition, overtime of 80 h or more per month is regarded as a benchmark for sudden death from overwork because it significantly increases the risk of developing cardiovascular disease (Araki and Iwasaki, 2005; Takahashi, 2019). However, according to the TALIS report, teachers in Japan work an average of 56 h per week, which results in 16 h of overtime per week and a cumulative average of 64 h of overtime per month (Organization for Economic Cooperation and Development [OECD], 2018). Because these circumstances imply that many teachers are working overtime beyond the benchmark for death from overwork, the impact of long working hours on teachers’ health has gained attention in Japan.

Several models of teachers’ stress have been presented by researchers, mainly in Europe and the United States (DeFrank and Stroup, 1989; Borg et al., 1991; Bottiani et al., 2019). Over 30 years ago, Borg et al. (1991) investigated teacher stress among primary school teachers and found that teachers’ occupational stress consisted of four aspects: pupil misbehavior, time/resource difficulties, professional recognition needs, and poor relationships. Interestingly, time management difficulty forms part of the model of occupational stress among teachers, even in Western countries (Borg et al., 1991). Over the last decade, it has been reported that long working hours are associated with depressive symptoms, psychological distress, or insomnia in Japanese teachers (Bannai et al., 2015a,b; Nomoto et al., 2015; Hori et al., 2020). Long working hours shorten sleep duration and reduce the rest needed to recover from fatigue, which can lead to serious health problems. Furthermore, long working hours not only cause insufficient sleep, prevent recovery from fatigue, and lead to chronic insomnia and/or psychosomatic complaints (von Bonsdorff et al., 2017; Nagaya et al., 2018; Ogawa et al., 2018), but may also reduce communication among faculty members and contribute to a decline in social support.

Conversely, there are various positions and roles within the teaching profession such as principal, tenured teacher, or nursing teacher. The work of these individuals is diverse, and they may have different ways of experiencing occupational stress. However, to the best our knowledge, no reports have examined the relationship between long working hours and stress responses across job titles or professions. Moreover, there are no reports on the results of a nationwide comparative study on the working hours of Japanese teachers. Therefore, the purpose of this study was to investigate the working hours of public junior high school teachers in Japan by job title, and to clarify the relationship between long working hours and psychological and physical stress responses from the results of a nationwide survey. First, we hypothesized that teachers’ working hours would differ depending on their job title. Second, we predicted that teachers in positions with long working hours would have a close relationship between working hours and stress responses.

## Materials and Methods

We used data from the Stress Check Program conducted between June and December 2018 by the Public School Teachers’ Mutual Aid Association on behalf of the Board of Education of each local self-government, such as prefecture, city, and town. The survey was conducted through a web-based questionnaire related to occupational stress, and assessed participant characteristics, job employment status, self-evaluated working hours per day, and work-related stress. The Stress Check Program was introduced by the Japanese government in 2015 following the revision of the Occupational Health and Safety Law in 2014, and requires implementation once a year in workplaces with 50 or more employees (Kawakami and Tsutsumi, 2016). The main purpose of the Stress Check Program is to prevent the occurrence of mental health problems. It is thought that by understanding the level of stress among employees, encouraging them to become aware of their own stress, and improving stressful work environments to create comfortable workplaces, the occurrence of mental health problems can be prevented (Kawakami and Tsutsumi, 2016).

We obtained information regarding sex, age, the number of years of continuous employment at the current school, responsibility for taking charge of the class, and job title (principal, vice-principal, tenured teacher, fixed-term teacher, nursing teacher, diet, and nutrition teacher).

The participants were 59,278 teachers and clerical workers working at junior high schools in 40 out of the 46 prefectures in Japan. Of these, 54,772 teachers with no missing data (female = 23,504, 42.9%) were included in the statistical analysis, excluding clerical workers. The percentage of eligible data was 92.4%. Of the teachers included in the analysis, 2,475 were principals (4.5%), 2,865 were vice-principals (5.2%), 40,869 were tenured teachers (74.6%), 5,442 were fixed-term teachers (9.9%), 2,609 were nursing teachers (4.8%), and 512 were diet and nutrition teachers (0.9%). Participating teachers belonged to 2,959 public junior high schools. In the Japanese education system, the designations of principal and vice-principal are administrative positions. In public schools, tenured teachers are registered teachers who have been accepted after passing the prefectural examination for teaching positions. Fixed-term teachers are generally appointed on a temporary basis and their contracts need to be renewed annually. A nursing teacher is a teacher who provides not only first aid in the nurse’s office but also advice on health management for the entire school. Their main duties include administering first aid to students who are injured or sick, conducting water and air tests, providing education on illness and injury prevention, managing health check-ups, and providing health room consultation. Diet and nutrition teachers are responsible for the operation of the school lunch program. They also provide students with accurate knowledge about nutrition and dietary habits.

The Ethics Committee of the Kinki Central Hospital of the Mutual Aid Association of Public School Teachers reviewed and approved the research aims, designs, and procedures of the internet-based survey (Approval number: 412). This study used existing data for the research. These data were already completely anonymized and untraceable; they will never be used to identify personal information. In addition, the subjects used their discretion and willingly responded to the questions, and the questions did not cause psychological distress to the subjects. Therefore, the ethics committee judged that informed consent was not required for this study.

### Measurements

#### Working Hours

We collected data about the number of working hours per day of the previous month, with seven response options as follows: (1) less than 8 h, (2) 8 to 9 h, (3) 9 to 10 h, (4) 10 to 11 h, (5) 11 to 12 h, (6) 12 to 13 h, and (7) 13 h or more.

#### Psychological and Physical Stress Responses

We used the Brief Job Stress Questionnaire (BJSQ) to assess whether the teachers were experiencing high levels of stress. The BJSQ is widely used in the field of occupational health in Japan and is an established questionnaire to identify high-stress workers (Wada et al., 2013; Inoue et al., 2014). The BJSQ has also been shown to have adequate reliability, internal consistency, and validity (Shimomitsu et al., 1999). It is a 57-item scale that is designed to assess the following three aspects of work: psychological job demands and job control (17 items), psychological and physical stress responses (29 items), and buffering factors such as social support at the workplace (11 items). In the present study, the total score of the psychological and physical stress responses (29 items) was used as an indicator of high-stress workers. Each item is rated on a four-point Likert-type scale (1 = almost never, 2 = sometimes, 3 = often, 4 = almost always), with higher scores indicating higher stress. In this study, scores could range from 29 to 116. Of the 29 items, 18 were concerning psychological stress responses requiring responses on the following five dimensions: liveliness (3 items; e.g., “I have been very active”), irritability (3 items; e.g., “I have been felt irritable”), fatigue (3 items; e.g., “I have been extremely tired”), anxiety (3 items; e.g., “I have felt worried or insecure”), and depression (6 items; e.g., “I have been depressed”). The physical stress response is assessed by 11 questions on physical complaints (e.g., “I have felt dizzy”). The total score of psychological and physical stress responses exhibited high internal consistency (Cronbach’s α = 0.90).

### Statistical Analysis

Percentages were computed for categorical variables. Concerning the variable of characteristics, age was grouped into the following categories: (1) 29 years old and below, (2) 30 to 39 years, (3) 40 to 49 years, (4) 50 to 59 years, and (5) 60 years and above. We divided the participants into two groups based on the number of years of continuous employment at the current school as follows: (1) less than 3 years, and (2) more than 3 years.

The chi-square test was used to compare the percentages between the groups. For the between-group comparisons in this study, we calculated the Cramér’s *V* to evaluate the effect size. Cramér’s *V* can be classified into weak (less than 0.1), moderate (0.1 to 0.3), and strong (0.3 or more) effect sizes.

In the statistical analysis, the stress response score was divided into quartiles and the group with the highest score was defined as the high-stress group, according to the classification protocol in a previous study (Wada et al., 2013). Multiple logistic regression analysis was conducted to examine the relationship between the hours of work per day and psychological and physical stress responses, adjusting for the effects of sex, age, classroom teacher status, and years of working at the current school. Due to the small number of teachers working less than 8 h, the teachers working less than 8 and 9 h were combined into a group for the multivariate analysis.

Statistical analyses were performed using IBM SPSS Statistics for Windows, version 23 (IBM Corp., Armonk, NY, United States), and *p* < 0.05 was considered statistically significant (two-tailed).

## Results

Table 1 shows the characteristics of the participants, and Table 2 presents the results of comparing the work hours per day across job titles of public junior high school teachers. Table 3 shows the results of the relationship between the number of working hours and physical and mental stress responses of each job tile of public junior high school teachers.

**TABLE 1:** Characteristics of junior high school teachers.

Demographic characteristics	*n*	%
**Sex**		
Male	31,268	57.1%
Female	23,504	42.9%
**Age**		
≤29	10,728	19.6%
30–39	12,195	22.3%
40–49	11,556	21.1%
50–59	16,957	31.0%
≥60	3,336	6.1%
**Number of years worked in same school**		
<3 years	23,933	65.0%
≥3	12,908	35.0%
**Job title in school**		
Principal	2,475	4.5%
Vice-principal	2,865	5.2%
Tenured teacher	40,869	74.6%
Fixed-term teacher	5,442	9.9%
Nursing teacher	2,609	4.8%
Diet and nutrition teacher	512	0.9%
**Taking charge of the class**		
Yes	26,636	48.6%
No	28,136	51.4%
**Working hours, per day**		
Less than 8 h	1,995	3.6%
8 to less than 9 h	3,234	5.9%
9 to less than 10 h	8,113	14.8%
10 to less than 11 h	8,824	16.1%
11 to less than 12 h	12,252	22.4%
12 to less than 13 h	10,893	19.9%
13 h and more	9,461	17.3%

**TABLE 2:** Comparisons of working hours per day by job title in junior high school teachers.

	Principal		Vice-principal		Tenured teacher		Fixed-term teacher		Nursing teacher		Diet and nutrition teacher		Total				
	*n*	%	*n*	%	*n*	%	*n*	%	*n*	%	*n*	%	*n*	%	χ^2^	*p*	Effect size
**Sex**																	
Male	2,307	93.2%	2,543	88.8%	23,692	58.0%	2,693	49.5%	15	0.6%	18	3.5%	31,268	57.1%			
Female	168	6.8%	322	11.2%	17,177	42.0%	2,749	50.5%	2,594	99.4%	494	96.5%	23,504	42.9%			
**Age**																	
≤49	11	0.4%	400	14.0%	27,809	68.0%	4,364	80.2%	1,518	58.2%	377	73.6%	34,479	63.0%			
50–59	2,007	81.1%	2,367	82.6%	10,951	26.8%	586	10.8%	927	35.5%	119	23.2%	16,957	31.0%			
≥60	457	18.5%	98	3.4%	2,109	5.2%	492	9.0%	164	6.3%	16	3.1%	3,336	6.1%			
**Hours of working per day**																	
Less than 8 h	57	2.3%	28	1.0%	1,126	2.8%	659	12.1%	89	3.4%	36	7.0%	1,995	3.6%	5295.8	<0.001	0.14
8 to less than 9 h	265	10.7%	44	1.5%	2,028	5.0%	393	7.2%	398	15.3%	106	20.7%	3,234	5.9%			
9 to less than 10 h	659	26.6%	151	5.3%	5,546	13.6%	810	14.9%	809	31.0%	138	27.0%	8,113	14.8%			
10 to less than 11 h	571	23.1%	270	9.4%	6,372	15.6%	841	15.5%	659	25.3%	111	21.7%	8,824	16.1%			
11 to less than 12 h	569	23.0%	540	18.8%	9,511	23.3%	1,086	20.0%	470	18.0%	76	14.8%	12,252	22.4%			
12 to less than 13 h	279	11.3%	924	32.3%	8,600	21.0%	922	16.9%	136	5.2%	32	6.3%	10,893	19.9%			
13 h and more	75	3.0%	908	31.7%	7,686	18.8%	731	13.4%	48	1.8%	13	2.5%	9,461	17.3%			

*Effect size was calculated by Cramér’s V.*

**TABLE 3:** Relationship between working hours per day and psychological and physical stress responses, in public junior high school teachers.

	Principal			Vice-principal			Tenured teacher		
	OR	95% CI lower – upper	*p*	OR	95% CI lower – upper	*p*	OR	95% CI lower – upper	*p*
Sex (Ref. Male)	3.16	1.90 – 5.25	<0.001***	1.26	0.88 – 1.79	0.201	1.58	1.50 – 1.67	<0.001***
Age	0.94	0.89 – 1.00	0.044*	0.97	0.94 – 1.00	0.088	1.00	1.00 – 1.01	0.030*
Taking charge of the class (Ref. No)	0.31	0.03 – 3.01	0.315	0.33	0.04 – 2.61	0.291	1.09	1.03 – 1.16	0.003**
Number of years worked in same school (Ref. <3 years)	1.37	0.89 – 2.11	0.148	1.37	0.96 – 1.94	0.079	0.93	0.88 – 0.99	0.017*
**Working hours, per day (Ref. <9 h)**									
9 to less than 10 h	0.89	0.46 – 1.72	0.736	0.53	0.20 – 1.37	0.188	1.12	0.98 – 1.28	0.091
10 to less than 11 h	1.37	0.72 – 2.61	0.335	0.71	0.32 – 1.61	0.418	1.25	1.10 – 1.42	0.001**
11 to less than 12 h	1.16	0.61 – 2.20	0.652	0.73	0.35 – 1.56	0.422	1.47	1.30 – 1.66	<0.001***
12 to less than 13 h	3.36	1.77 – 6.38	<0.001***	0.77	0.37 – 1.59	0.485	1.64	1.45 – 1.85	<0.001***
13 h and more	2.47	0.96 – 6.39	0.062	1.32	0.64 – 2.68	0.452	2.43	2.14 – 2.75	<0.001***

	**Fixed–term teacher**			**Nursing teacher**			**Diet and nutrition teacher**		
	**OR**	**95% CI lower – upper**	***p***	**OR**	**95% CI lower – upper**	***p***	**OR**	**95% CI lower – upper**	***p***

Sex (Ref. Male)	1.56	1.33 – 1.83	<0.001***	1.42	0.33 – 6.06	0.635	0.50	0.16 – 1.53	0.227
Age	0.99	0.98 – 0.99	<0.001***	0.98	0.97 – 0.99	0.001**	0.99	0.97 – 1.01	0.530
Taking charge of the class (Ref. No)	1.09	0.93 – 1.29	0.297	2.16	0.62 – 7.48	0.226			
Number of years worked in same school (Ref. <3 years)	0.86	0.64 – 1.15	0.309	1.03	0.80 – 1.32	0.827	1.66	1.02 – 2.70	0.041*
**Working hours, per day (Ref. <9 h)**									
9 to less than 10 h	1.59	1.18 – 2.14	0.002**	1.22	0.85 – 1.74	0.285	1.95	1.02 – 3.74	0.043*
10 to less than 11 h	1.88	1.40 – 2.52	<0.001***	1.18	0.81 – 1.71	0.385	1.86	0.96 – 3.60	0.066
11 to less than 12 h	1.65	1.24 – 2.20	0.001**	1.95	1.34 – 2.84	0.001**	2.12	1.00 – 4.49	0.049*
12 to less than 13 h	2.64	1.98 – 3.51	<0.001***	2.95	1.79 – 4.86	<0.001***	2.04	0.79 – 5.27	0.142
13 h and more	3.60	2.69 – 4.82	<0.001***	1.71	0.71 – 4.11	0.229	2.48	0.54 – 11.37	0.241

– OR: Odds ratio; 95% CI: 95% of confidence interval; *p < 0.05; **p < 0.01; ***p < 0.001.

As shown in Table 1, it was most common for teachers to work more than 11 h but less than 12 h a day (22.4%). Teachers who worked between 12 and 13 h a day comprised the second-largest group, and those who worked more than 13 h a day comprised the third-largest group. Teachers who worked more than 11 h a day accounted for 59.6% of the total.

Table 2 shows the results of the comparison of working hours by job title. The results revealed statistically significant differences in the length of daily work hours by job title (χ^2^ (30) = 5295.8, *p* < 0.001). Cramér’s *V* was 0.14, indicating a moderate effect size of difference. First, vice-principals generally worked longer hours, with 31.7% of them working more than 13 h a day. Second, tenured teachers worked long hours, with 18.8% of them working more than 13 h a day. Third, 13.4% of fixed-term teachers worked long hours. On the other hand, there were also statistically significant differences in sex (χ^2^ (5) = 6634.1, *p* < 0.001, Cramér’s *V* = 0.35) and age (χ^2^ (20) = 11732.2, *p* < 0.001, Cramér’s *V* = 0.23), with principals and vice-principals more likely to be male and over 50 years old.

Since there was a significant difference in the number of working hours by job title, sex and age, multiple logistic regression analysis was conducted to determine the association between the number of working hours and psychological and physical stress responses, adjusting for the effects of sex, age, and other variables. The results of the multiple logistic regression analysis are shown in Table 3, which aimed to examine the relationship between work hours and psychological and physical stress responses. For principals, sex (odds ratio [OR] = 3.16 with 95% confidence interval [CI] = 1.90–5.25, *p* < 0.001), age (OR = 0.94, CI = 0.89–1.00, *p* = 0.044), and working more than 12 h and less than 13 h (OR = 3.36, CI = 1.77–6.38, *p* < 0.001) were associated with psychological and physical stress responses.

Regarding vice-principals, there was no statistically significant relation between working hours and psychological and physical stress responses.

Regarding tenured teachers, sex (OR = 1.58, CI = 1.50–1.67, *p* < 0.001), age (OR = 1.00, CI = 1.00–1.01, *p* = 0.030), taking charge of the class (OR = 1.09, CI = 1.03–1.16, *p* = 0.003), number of working years in the same school (OR = 0.93, CI = 0.88–0.99, *p* = 0.017), and working hours were associated with psychological and physical stress responses. In particular, working 10 to 11 h (OR = 1.25, CI = 1.10–1.42, *p* = 0.001), 11 to 12 h (OR = 1.47, CI = 1.30–1.66, *p* < 0.001), 12 to 13 h (OR = 1.64, CI = 1.45–1.85, *p* < 0.001), and 13 h or more (OR = 2.43, CI = 2.14–2.75, *p* < 0.001) were significantly associated with psychological and physical stress responses.

For fixed-term teachers, sex (OR = 1.56, CI = 1.33–1.83, *p* < 0.001), age (OR = 0.99, CI = 0.98–0.99, *p* < 0.001), and working 9 to 10 h (OR = 1.59, CI = 1.18–2.14, *p* = 0.002), 10 to 11 h (OR = 1.88, CI = 1.40–2.52, *p* < 0.001), 11 to 12 h (OR = 1.65, CI = 1.24–2.20, *p* = 0.001), 12 to 13 h (OR = 2.64, CI = 1.98–3.51, *p* < 0.001), and 13 h or more (OR = 3.60, CI = 2.69–4.82, *p* < 0.001) were related with psychological and physical stress responses.

For nursing teachers, age (OR = 0.98, CI = 0.97–0.99, *p* = 0.001), working 11 to 12 h (OR = 1.95, CI = 1.34–2.84, *p* = 0.001), and 12 to 13 h (OR = 2.95, CI = 1.79–4.86, *p* < 0.001) were associated with psychological and physical stress responses.

As for diet and nutrition teachers, the number of years of working in the same school (OR = 1.66, CI = 1.02–2.70, *p* = 0.041), working 9 to 10 h (OR = 1.95, CI = 1.02–3.74, *p* = 0.043), and 11 to 12 h (OR = 2.12, CI = 1.00–4.49, *p* = 0.049) were associated with psychological and physical stress responses.

In the association between working hours and stress response, the odds ratio tended to increase with longer working hours compared to those who worked less than 9 h for all job titles except vice-principal.

## Discussion

The purpose of this study was to investigate the working hours of public junior high school teachers through a nationwide survey. In addition, we explored the relationship between long working hours and psychological and physical stress responses across job titles.

### Working Hours of Japanese Public Junior High School Teachers

In Japan, overtime work of more than 720 h per year is illegal, with penalties related to Japan’s Labor Standards Law. However, this law did not apply to teachers in public schools as of 2020 (Ministry of Justice, Japan, 2012). Working more than 11 h a day is one of the benchmarks for long working hours, as it is equivalent to working more than 720 h overtime a year if continued for a year. In addition, Hayashi et al. (2019) revealed that overtime work of 60 h or more per month significantly increases the risk of developing cardiovascular disease (Hayashi et al., 2019). However, 59.6% of the teachers worked for 11 h or more per day (overtime of more than 3 h per day). Furthermore, overtime work of more than 80 h per month is considered a benchmark for death from overwork, because it increases the incidence of acute myocardial infraction (Araki and Iwasaki, 2005). A total of 37.2% teachers were working more than 12 h or more per day (overtime of more than 4 h per day). Takahashi (2019) conducted analyses of compensated claims for overwork-related cerebrovascular and cardiovascular disease and mental disorders that were recognized between 2010 and 2015, and revealed that long working hours were the principal factor for overwork-related cerebrovascular and cardiovascular disease. Long working hours reduce the amount of sleep and rest needed to recover from fatigue, which can lead to chronic sympathetic hyperactivity, which can consequently lead to serious health problems. Nevertheless, in the field of education, necessary occupational safety and health policies were not implemented until 2020. Additionally, working hours differed by job title: 64.0% vice-principals, 39.8% tenured teachers, 30.3% fixed-term teachers, 14.3% principals, 8.8% diet and nutrition teachers, and 7.0% nursing teachers worked 4 h or more overtime per day. Specifically, our results suggest that the teachers with the position of vice-principal are at high-risk of occupational health problems. This study also revealed the problematic situation of junior high school teachers working overtime in Japan.

### Long Working Hours and Stress Responses

The associations of working hours with stress response were examined across job titles; the number of working hours was significantly related to high stress response. Somatic symptoms in teachers are often caused by long working hours and other unfavorable working conditions (Van der Hulst, 2003). It has also been reported that long working hours among teachers is associated with mental health problems such as depressive symptoms, psychological distress, and insomnia (Bannai et al., 2015a,b; Nomoto et al., 2015; Hori et al., 2020). However, the present study revealed that the relationship between long working hours and stress differs depending on the teacher’s position. Long working hours among tenured teachers and fixed-term teachers are particularly closely associated with their stress responses; working 12 h or more per day (overtime of 4 h per day) among tenured teachers was associated with a 1.64-fold higher risk of being in the high-stress group compared to those working less than 9 h per day. Additionally, the number of fixed-term teachers who worked 12 h or more per day was 2.64 times higher in the high-stress group compared to those who worked less than 9 h per day. The duties of a teacher in Japan are diverse and include not only academic teaching, but also providing guidance regarding students’ daily lives, handling students who are absent from school, guiding students on their future career paths, and instructing students on club activities outside of the classroom. This large number of work responsibilities is thought to be the cause of teachers’ long working hours. In addition, fixed-term teachers must work at different schools every few years, because their contracts are renewed annually. They need to receive a good evaluation in a short period of time to be rehired the following year. Besides, since they are non-permanent positions, their wages are lower than those of tenured teachers. Worry about employment for the following year and poor employment conditions may contribute to their psychological strain, increasing their stress responses. In recent years, the number of fixed-term teachers has been on the rise in Japan, and the worsening employment conditions and heavy workload may be causing unclear career prospects among candidates for teaching positions, and keeping talented people away from the field of education. Stress responses of fixed-term teachers has been an ignored issue, and may become an important research topic to be addressed in the future.

Because the working hours of vice-principals were longer than teachers in other positions, we predicted that long working hours would be associated with higher stress responses in vice-principals as well. However, contrary to our expectation, there was no statistically significant relationship between working hours and stress responses in vice-principals. Workaholism is a possible explanation for why working hours and stress response were not related in this case. Workaholism is defined as a compulsive devotion to work that significantly impairs other areas of an individual’s life (Selinger, 2007). Teaching is one of the occupations most prone to struggling with workaholism (Reysen et al., 2014), and vice-principals who work excessively long hours possibly tend to have difficulty recognizing their own stress due to workaholic symptoms. Moreover, it is possible that vice-principals themselves no longer regard long working hours as a problem, because overtime work among vice-principals has become extremely common. However, we believe that the lack of association between working hours and stress responses of vice-principals does not prove that there is no harm to health. The reason for this is the fact that the number of demotions based on the requests of vice-principals themselves has been increasing in recent years (Ministry of Education, Culture, Sports, Science and Technology [MEXT], 2017). Additionally, vice-principals are usually older than tenured teachers and fixed-term teachers, and aging is an important risk factor for myocardial infarction and cerebrovascular disease. Therefore, it is necessary to mention that the current situation of vice-principals engaging in excessive long work hours without awareness of overwork and a lack of sleep is problematic.

For teachers such as principals, nursing teachers, and diet and nutrition teachers, there was a significant association between working hours and stress responses. However, there was no clear trend that the frequency of teachers with high stress increased as working hours increased. There are some possible reasons why working hours were only marginally associated with stress response. First, the data may be influenced by the fact that the percentage of teachers who work long hours is smaller than that of tenured and fixed-term teachers. Second, it may be related to the fact that teachers in each of these positions have a definitive job description. Principals are responsible for school administration and children’s safety, school nurses for children’s health and hygiene, and nutrition teachers for nutritional guidance. Being able to contribute their expertise in their work is considered to be a protective factor against occupational stress. Third, several previous studies have reported the influence of buffering factors on work stress (Hultell and Gustavsson, 2011; Boström et al., 2019; Slišković et al., 2019). Worthwhile and motivational attitude may also moderate the relationship between the number of work hours and stress responses. These factors that are potentially associated with occupational stress should be considered in the future while exploring the relationship between working hours and stress responses.

### Limitations

We mention several limitations of this study. First, because a cross-sectional research design was adopted, we cannot deduce causality of the relationship between long working hours and stress responses. Second, we believe that there are some variables that moderate the relationship between working hour and stress responses. Previous research revealed that job control and social support moderate teachers’ well-being (Ibrahim et al., 2021). In addition to these variables, work engagement (Hultell and Gustavsson, 2011), work environment (Boström et al., 2019), and social support (Steptoe, 2000; Slišković et al., 2019) might be also related to stress responses among teachers. These factors may modify the relationship between long working hours and stress responses. If possible, future studies should examine a model which incorporates these variables applying structural equation modeling. Therefore, more research is needed to better understand the factors associated with teachers’ work environments, as well as factors that buffer the stress responses. Research on how to improve the industry hygiene of teachers would be a worthy topic for future study.

### Conclusion

In summary, this research explored the working environment and the relationship between long working hours and stress responses in public junior high school teachers in Japan. Results revealed the problematic situation of long working hours for these teachers. Specifically, vice-principals work the longest hours among teachers, which might be a hidden problem that is often overlooked, even by themselves. Furthermore, working hours of tenured and fixed-term teachers are closely related to stress responses requiring effective and sustainable solutions. For sustainable action, it is essential to investigate the causes of long working hours and analyze factors related to stress responses in tenured and fixed-term teachers in the future.

## Data Availability Statement

The datasets presented in this article are not readily available because of participant privacy. Requests to access the datasets should be directed to SY, yamamura_s@kich.itami.hyogo.jp.

## Ethics Statement

The studies involving human participants were reviewed and approved by the Ethics Committee of the Kinki Central Hospital of the Mutual Aid Association of Public School Teachers (Approval number: 412). The ethics committee waived the requirement of written informed consent for participation.

## Author Contributions

MM and SY conceptualized the study. MM analyzed the data and wrote the manuscript. SY supervised the study. Both authors contributed to manuscript revision and approved the submitted version.

## Conflict of Interest

The authors declare that the research was conducted in the absence of any commercial or financial relationships that could be construed as a potential conflict of interest.

## Publisher’s Note

All claims expressed in this article are solely those of the authors and do not necessarily represent those of their affiliated organizations, or those of the publisher, the editors and the reviewers. Any product that may be evaluated in this article, or claim that may be made by its manufacturer, is not guaranteed or endorsed by the publisher.
